# Commentary: 3-Iodothyronamine Reduces Insulin Secretion In Vitro via a Mitochondrial Mechanism

**DOI:** 10.3389/fendo.2018.00057

**Published:** 2018-02-28

**Authors:** Annunziatina Laurino, Laura Raimondi

**Affiliations:** ^1^Section of Pharmacology and Toxicology, Department of Psychology, Neurology, Drug Sciences, Health of the Child, Pharmacology, University of Florence, Florence, Italy

**Keywords:** 3-iodothyronamine, 3-iodothyroacetic acid, hyperglycemia, diabetes, amine oxidases, amine oxidase inhibitors

Lehmphul et al. report the effect of 3-iodothyronamine in reducing insulin release in a model of immortalized pancreatic β-cells. Notwithstanding the simplified β-cell model used, this article offers an opportunity to reconsider, possibly under a new light, an old issue of research, which excited people working on amine oxidases (AOs) in the last 20 years. Toward this aim, we would like to propose some points of reflection to the scientific community working on 3-iodothyronamine and thyroid hormone metabolites:

1. the paper indicates that 3-iodothyronamine reduces insulin release with a mechanism mediated, at least in part, by its oxidative metabolite, the 3-iodothyroacetic acid, produced by mitochondrial monoamine oxidase (MAOs), type B (MAO-B) activity. This finding, confirming our observations and hypothesis on the role of 3-iodothyronamine as a source of active metabolites (1, 2), demonstrates for the first time that 3-iodothyronamine is a substrate for MAO-B, the MAO isoform in search of substrates and of functions;
2. the degradation of 3-iodothyronamine by MAO-B, with production of the corresponding aldehyde and hydrogen peroxide (H_2_O_2_), potentially represents a self-standing mechanism independently of 3-iodothyronamine receptor activation on pancreatic cells.

Amine oxidases are a heterogeneous class of enzymes, including MAOs (type A and B) and semicarbazide-sensitive amine oxidases (SSAOs). While MAOs are ubiquitous enzymes, being linked to the outer mitochondrial membrane (active site facing the cytoplasm), plasma membrane SSAOs can have selective and species-specific tissue/cell expression. In addition, MAOs and SSAOs are distinguishable by inhibitor sensitivity, substrate selectivity and affinity, and subcellular localization. Noradrenaline and serotonin are among MAO-A substrates, dopamine and other trace amines, including tyramine and β-phenylethylamine, are MAO-A, B, and SSAO substrates. Up to now, direct evidence that 3-iodothyronamine is a substrate for MAO-A is lacking. However, now we know that 3-iodothyroanime is a substrate for MAO-B.

## AO Catalysis: A Pro-Oxidant Source for Diabetes Complications

The oxidative deamination carried out by AOs produces substrate-derived aldehydes, H_2_O_2_, and ammonia. Aldehydes and H_2_O_2_ are well known pro-oxidant compounds scavenged by aldehyde dehydrogenase(s) and catalase activities, respectively, to the corresponding carboxylic acid and water. If produced outside the cell by SSAO activity, H_2_O_2_ may have two fates: to enter cells or to remain outside cells. Both conditions can be a trigger for intra- or extracellular milieu oxidation with the latter compartmentalization as a pathogenic mechanistic event generating micro- and macrovascular damage. Aldehydes from SSAO catalysis can generate carbonylation of extracellular proteins as their scavenging to the corresponding carboxylic acid can only occur intracellularly.

If produced, by MAO activities, H_2_O_2_ may be scavenged by catalase or freely diffused throughout organelle membranes, generating a potential localized change in the redox state, a condition recognized as one among the main pathogenic events triggering the pancreatic dysfunction, insulin resistance, and long-term deleterious effects in exhausting cell/tissue antioxidant defenses. Furthermore, insulin-target cells were described as a preferential site for SSAO and MAO expression (2, 3), with their activities further increased in hyperglycemia (4) as well as in hypertension, obesity, and in other cardiovascular diseases (2–5), likely as a consequence of increased levels of pro-inflammatory signals (6).

## AO Catalysis: The Hypoglycemic and Insulin Mimetic Effects of AO Substrates

Hydrogen peroxide can also have beneficial signaling activities, including its capacity to activate the trafficking of GLUT4 in adipocytes and other insulin-sensitive cells. Several studies have highlighted the use of high concentrations of non-selective SSAO and MAO substrates in stimulating GLUT4 activity, thus reducing hyperglycemia and mimicking insulin effects, including adipocyte differentiation (7–9). On the other hand, SSAO substrate degradation was found to be a trigger for the generation of advanced glycation products (10). Therefore, whether AO inhibitors or substrates should be proposed for controlling diabetes thus remained an open issue (11).

## Protective Effects of AO Inhibition: Clinical and Experimental Evidence

Aminoguanidine, an inhibitor of SSAOs, is effective in reducing advanced glycation end products in diabetic patients and in experimental diabetes (12, 13). More interestingly, clinical and experimental evidence indicate that the beneficial effects of drug targeting angiotensin II cascade in preventing diabetes complications might include the control of MAO activities (14, 15), which may play a pathogenic role in different cardiomyopathies (5).

These evidence confirm the pro-oxidant and pro-inflammatory role for AO catalysis and an overall beneficial effect of reducing their activities.

## 3-Iodothyronamine: What’s New?

The novel fact is that 3-iodothyronamine (i) is a common endogenous substrate for MAO-B and SSAOs, (ii) its plasma levels increased in diabetic patients (16), and (iii) when administered to mice it induces hyperglycemia (central and/or peripheral effects) with a mechanism that remains to be clarified but dependent, at least in part, on MAO activity (17, 18). To note, we have collected evidence demonstrating that MAO activities are involved in the conversion of endogenous but also pharmacological administered 3-iodothyronamine into 3-iodothyroacetic acid (19). This latter result suggests that 3-iodothyroacetic acid/3-iodothyronamine might be homeostatically regulated *via* AO activities.

Even if it is not demonstrated yet, under conditions of hyperglycemia, the products of oxidative deamination of 3-iodothyronamine are expected to increase. Overall, hyperglycemia might reflect a condition of an unbalanced 3-iodothyronamine rate of synthesis and degradation, making available a great amount of the “pro-diabetic” 3-iodothyroacetic acid and pro-oxidant compounds, which can exacerbate diabetes and its complications.

**Figure 1:**
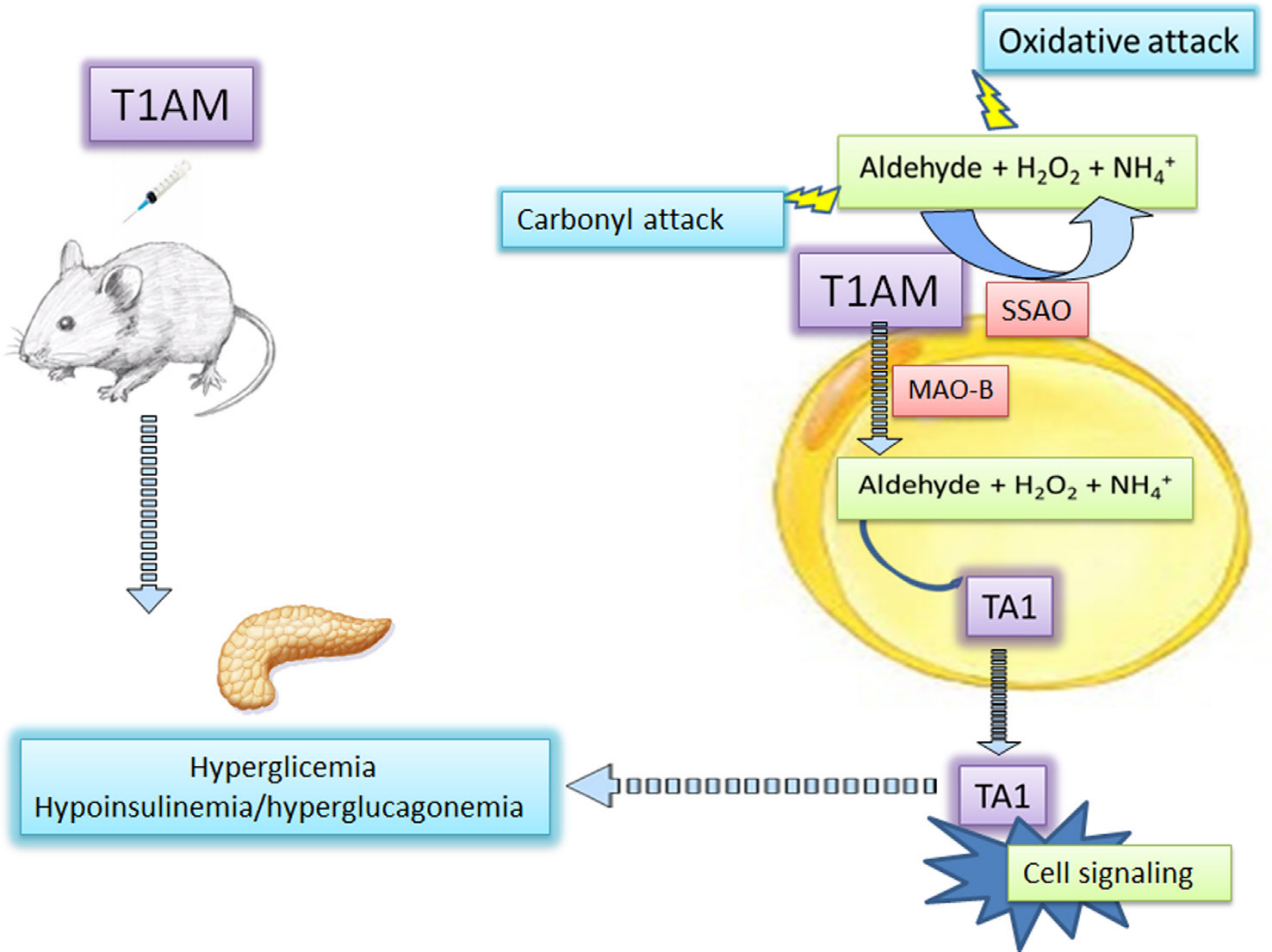
3-Iodothyronamine and hyperglycemia: the mediation of amine oxidases. 3-Iodothyronamine (T1AM) injected in mice induces hyperglycemia likely interacting at different pancreatic receptors promoting glucagon and reducing insulin release. At insulin-sensitive cells, including the pancreas, T1AM is converted into 3-iodotyroactic acid (TA1), the oxidative metabolite of T1AM by the activities of mitochondrial monoamine oxidase (MAOs), or semicarbazide-sensitive amine oxidases (SSAOs). TA1 can diffuse from cells and induce cell signaling activities and promoting hyperglycemia. The secondary product of amine oxidase activities, i.e., hydrogen peroxide (H_2_O_2_) and the aldehyde, may promote oxidative attack to cell components.

Since thyroid dysfunctions are a risk factor for diabetes and because 3-iodothyroacetic acid/3-iodothyronamine seems to be homeostatically regulated, the circle around AOs and hyperglycemia might be conclusively closed. Consequently, the measure of 3-iodothyroacetic acid/3-iodothyronamine plasma levels may have diagnostic relevance to predict the risk of hyperglycemia (Figure 1).

## Author Contributions

AL and LR wrote the paper and prepared the figure.

## Conflict of Interest Statement

The authors declare that the research was conducted in the absence of any commercial or financial relationships that could be construed as a potential conflict of interest.
